# Thyroid liposarcoma: a case report

**DOI:** 10.1515/iss-2021-0037

**Published:** 2022-10-14

**Authors:** Maxime Gerard, Alexander N. Flaris, Marco Demarchi, Ilies El Boukili, Laure Maillard, Françoise Borson-Chazot, Myriam Decaussin-Petrucci, Jean-christophe Lifante

**Affiliations:** Service de chirurgie endocrinienne, Hôpital Lyon Sud, Pierre Bénite, France; Department of Surgery, School of Medicine, Tulane University, New Orleans, LA, USA; RESHAPE Research on Healthcare Performance, INSERM U1290-UCBL 1, Domaine Rockefeller, 8 Avenue Rockefeller, Lyon, France

**Keywords:** sarcoma, thyroid liposarcoma, thyroid surgery

## Abstract

**Objectives:**

Thyroid liposarcoma is a rare tumor. Its low prevalence accounts for the scarcity of data in the literature, which consists mostly of small studies and case reports.

**Case presentation:**

We present the case of a 60 years old male with no past medical or past surgical history and presented with neck discomfort and a large left thyroid nodule. Thyroid ultrasound and CT scan were performed and confirmed the existence of a thyroid nodule most probably inside the left inferior thyroid lobe. In the posterior mediastinum, two fatty formations were found. To complete, an MRI was performed, showing a mixed lesion, of the lower neck and upper chest. The patient underwent an extended resection which consisted of an en bloc resection of the lesion (left thyroid lobectomy and isthmus resection) by an anterior transverse cervical incision and a sternotomy. Tracheal and laryngeal shaving and esophageal shaving with resection of the esophageal muscularis was performed as well. The pathological evaluation of the specimen showed a grade II dedifferentiated liposarcoma with an inflammatory component.

**Conclusions:**

Thyroid liposarcoma is a rare lesion of the thyroid. Its management requires an exhaustive workup followed by an en bloc resection of the lesion. Depending on the histology, postoperative radiation therapy may or may not be necessary.

## Introduction

Soft tissue sarcomas are tumors of mesenchymal origin that represent less than 1% of head and neck malignancies and less than 5% of sarcomas [1]. The most frequent histological type is liposarcoma which represents about 20% of all soft tissue sarcomas [2]. Dedifferentiated liposarcoma and well-differentiated liposarcoma are the most common types of liposarcoma and together account for 80% of liposarcomas [3]. The peak incidence of liposarcoma is during the 7th decade and involves mainly the limbs, the trunk and the retroperitoneum, rarely the mediastinum.

Thyroid liposarcoma is a rare tumor representing less than 1.5% of thyroid malignancies [4]. Its low prevalence accounts for the scarcity of data in the literature, which consists mostly of small studies and case reports. The diagnostic algorithm for thyroid liposarcomas is the same as the one used for thyroid nodules. Although there is no consensus in the literature on a comprehensive treatment plan for patients with thyroid sarcoma, surgery plays a central role in almost all cases. Adjuvant treatment has not been studied specifically in the management of this disease, but there is an emerging literature regarding the adjuvant treatment of soft tissue sarcomas of the head and neck.

In this article we present the case of a dedifferentiated liposarcoma of the thyroid in a 60-year-old male.

## Case presentation

The patient was a 60-year-old male with no past medical or past surgical history. He presented with neck discomfort and a large left thyroid nodule discovered on self-palpation.

A thyroid ultrasound showed that this nodule was intrathyroidal, measuring 4 cm in diameter and was classified as TIRADS 4B: slightly heterogeneous aspect, nodule thicker than wide. A non-contrast CT scan was then obtained in order to further delineate the anatomy of the area, as it was difficult to evaluate the relationship of the mass to the surrounding structures on ultrasound alone. The CT scan showed left thyroid lobe hypertrophy with extension of the lobe into the upper chest without evidence of invasion into the surrounding structures. There were also fatty formations in the upper and posterior mediastinum, displacing the trachea and the esophagus ventrally.

**Figure 1:**
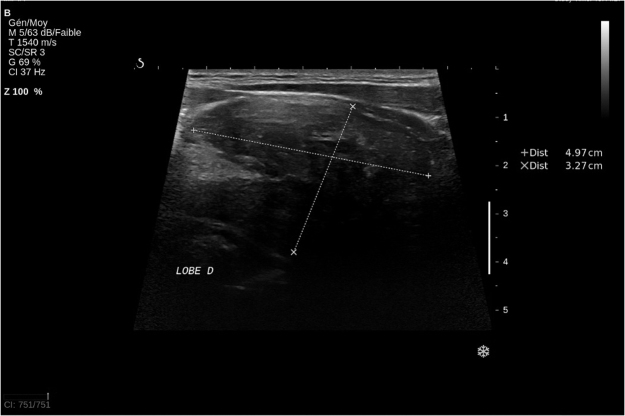
US examination of the thyroid.

A new thyroid ultrasound (Figure 1) was performed at the Lyon Sud Hospital by a radiologist familiar with thyroid imaging. This ultrasound showed a left cervical mass of a rather extra-thyroidal appearance according to the radiologist. Fine needle aspiration was performed. Pathology was suspicious for malignancy, without being able to determine the cancer type (anaplasic vs. sarcoma vs. papillary) because of the limited tissue quantity.

**Figure 2:**
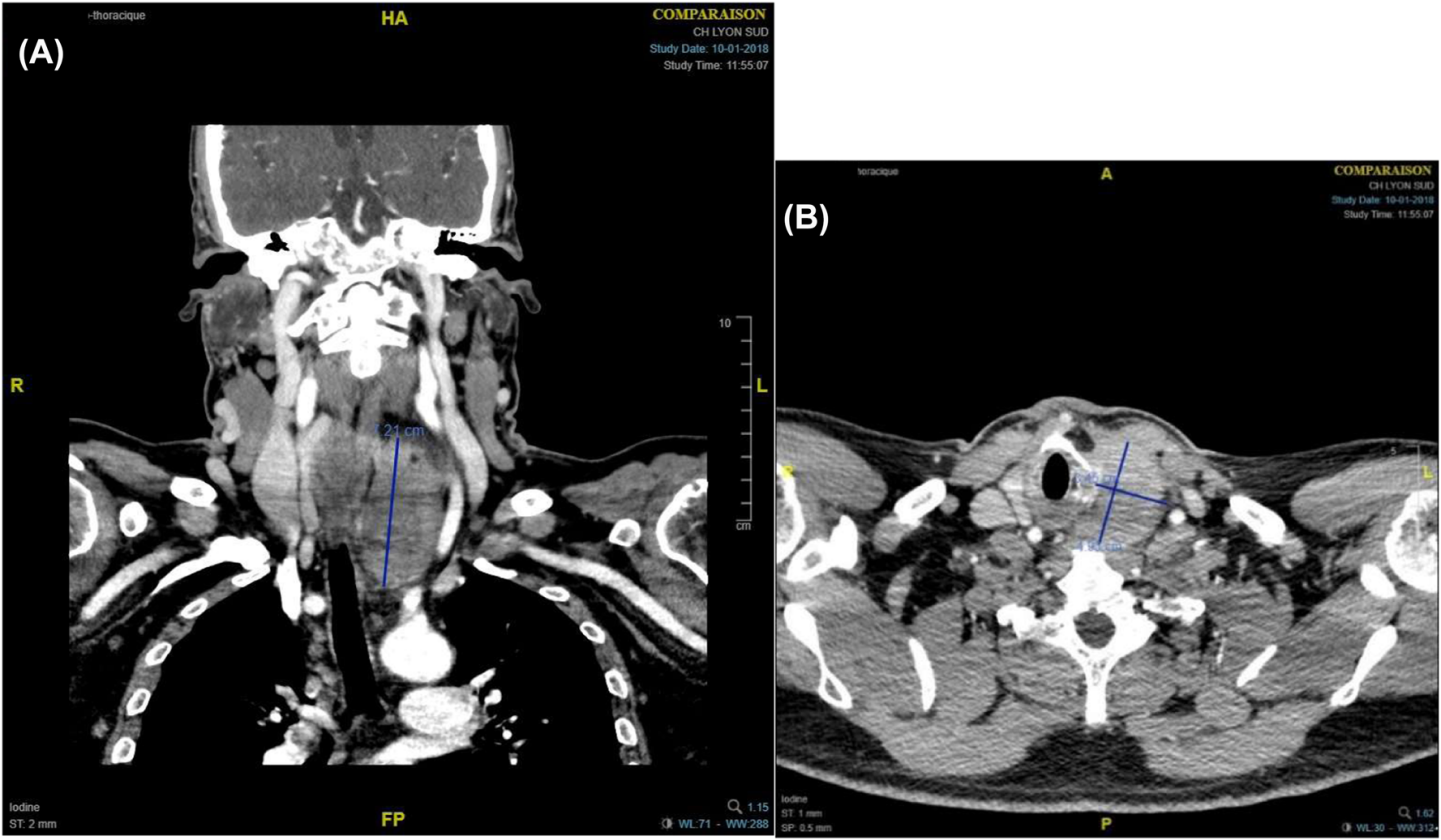
Second CT scan (with contrast). (A) Coronal view. (B) axial view.

A new CT scan was performed, this time with contrast. This second scan (Figure 2) confirmed the existence of a thyroid nodule most probably inside the left inferior thyroid lobe, measuring about 7 cm in height and 4.5 cm in diameter. There were no visible calcifications. The nodule was compressing the trachea resulting in a decrease of the trachea’s diameter. The large vessels were compressed but remained patent. In the posterior mediastinum, two fatty formations were found, measuring 30 × 37 mm and 40 × 49 mm. The density of the upper formation was −290 HU and that of the lower formation was between 70 and 80 HU. These two formations displaced the trachea and the esophagus ventrally.

Lab work did not provide any additional information. Thyroid function tests were normal, anti-thyroid antibodies were negative and calcitonin levels were normal.

**Figure 3:**
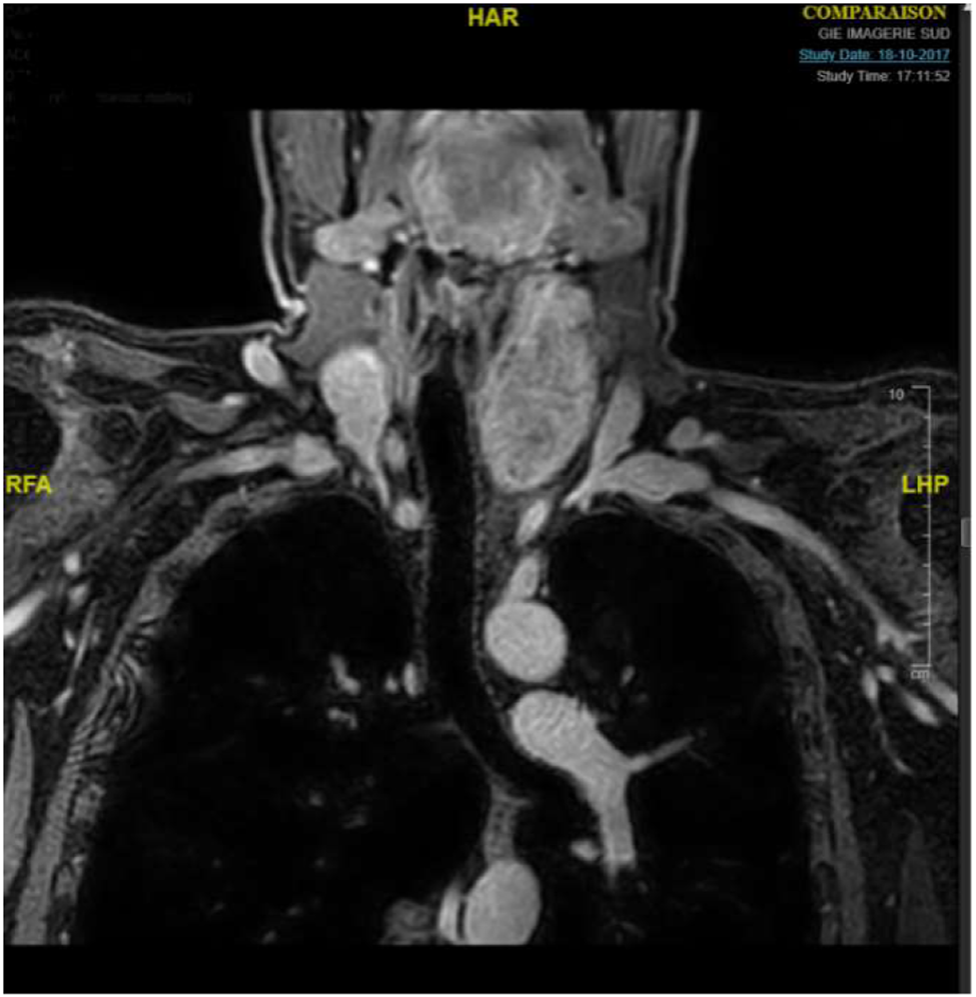
MRI of the left cervical lesion (axial view).

To complete the workup, an MRI was performed, showing a mixed lesion, of the lower neck and upper chest, extending over 13 cm in length with a cervical tissue component displacing posteriorly the thyroid and the carotid sheath, without any invasive criteria and a thoracic component which was mainly fatty, in contact with the esophagus, the common carotid artery and the left subclavian artery. The examination could not differentiate between teratoma and liposarcoma (Figure 3).

After evaluation by a multidisciplinary team, the patient underwent an extended resection of a liposarcoma of the neck with intra-thoracic extension. The procedure consisted of an en bloc resection of the lesion (left thyroid lobectomy and isthmus resection) by an anterior transverse cervical incision. A sternotomy was performed as well in order to access the thoracic portion of the lesion. Tracheal and laryngeal shaving and esophageal shaving with resection of the esophageal muscularis was performed as well. The left internal jugular vein and the left vagus nerve were sacrificed in order to guarantee an R0 resection. This resulted into a left recurrent laryngeal paralysis; however this was unavoidable in order to ensure negative margins.

The patient did well postoperatively and was discharged home on postoperative day (POD) 5. A CT scan was performed on POD7 for acute respiratory distress. The CT showed an esophageal leak. The patient was taken back for a washout and wide drainage. He did well afterwards and the leak eventually healed.

**Figure 4:**
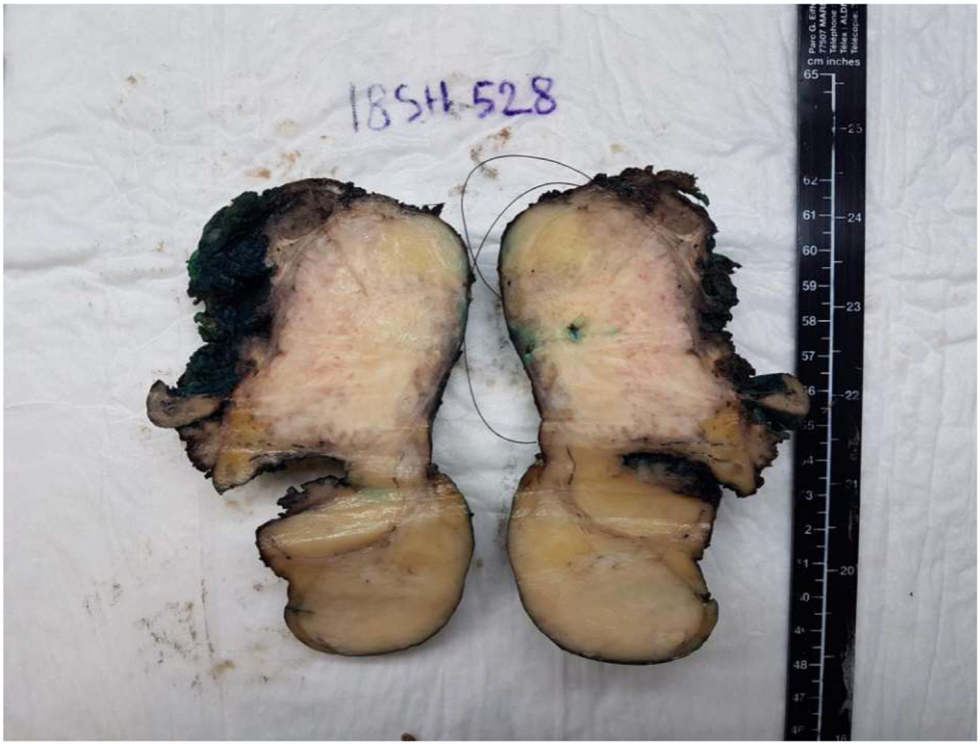
Pathology specimen: left thyroid lobectomy.

The pathological evaluation of the specimen was performed at a referral center and showed that the lesion was a grade II dedifferentiated liposarcoma with an inflammatory component (Figure 4) invading the thyroid and the strap muscles. Margins at the isthmus were positive on microscopic examination and negative at esophageal and trachea. The left jugular vein lymph nodes were negative.

A follow up CT scan did not show any recurrence neither at the thyroid nor at any distant sites. There was suspicion of a recurrence at the neck scar, however biopsies were negative. The patient was then treated with local radiation therapy, 60 Gy in 30 sessions.

The patient was finally seen at 18 months post-operatively. He was in good general condition and the CT scan did not show any progression of his disease. At 4 years postoperatively, the patient still does not have any local recurrence on imaging.

## Discussion

Liposarcomas are solid adipocytic tumors representing a heterogeneous group of lesions grouped into four main pathology groups by the WHO (atypical lipoma/well-differentiated liposarcoma, myxoid, pleomorphic, and dedifferentiated liposarcoma) [5, 6]. Thyroid sarcomas in general represent less than 2% of thyroid malignancies, and liposarcomas are even rarer lesions. A 2015 review of the literature included all of the thyroid sarcoma cases available in the literature. This review found a total of 142 patients with primary thyroid sarcoma. Among all these patients, only 7 (4.9%) liposarcomas were found [7]. A more recent review (2018) included all the cases of thyroid liposarcoma in the literature. This review found a total of 12 patients with liposarcoma [8]. Among these liposarcomas, there were three well-differentiated liposarcomas, three pleomorphic liposarcomas, three myxoid liposarcoma, and one dedifferentiated liposarcoma. Despite a growing literature, dedifferentiated liposarcoma remains a rare tumor for which there is no consensus on how to manage it.

The term “undifferentiated liposarcoma” was introduced by Evans in 1979 to define the morphologic progression of an atypical lipomatous tumor/well-differentiated liposarcoma to a non-lipogenic sarcoma [9]. Most often, the non-lipogenic component is high grade; however, the WHO classification recognizes the existence of cases in which the undifferentiated component is morphologically low grade [10]. Despite its typically high-grade morphology, dedifferentiated liposarcoma is much less aggressive than other types of high-grade pleomorphic sarcomas [11]. Differentiation is associated with a metastasis rate of 15–20%; however, mortality is more often related to uncontrolled local recurrence than metastatic spread. Because the time to recurrence appears to correlate with the extent of the initial resection, current treatment includes a wide surgical resection that should include adjacent viscera [12]. However, this resection should not be unreasonably extensive or lead to a morbidity that is greater than the benefit of the surgical resection. In our case, we decided to preserve the esophagus and the trachea. Intraoperatively, the esophagus seemed to be invaded at least at the level of the muscularis. After wide resection of the lesion – including the muscularis of the esophagus – pathology did not find any invasion of the muscularis. In case of a rather superficial lesion, resection of only the muscular layer is possible since the strength layer of the esophagus is the remaining mucosa. A French series of 21 patients with invasive thyroid cancer invading either the esophagus or the trachea showed that, for esophagus invasion, resecting the muscularis alone while leaving the deeper layers intact and then closing the defect primarily led to good results without esophageal fistulas [13]. On the trachea, it was decided to perform a shave excision, which consists of removing as much of the tumor as possible without damaging the trachea; this kind of excision is possible only in cases of superficial lesions of the trachea [14]. Even though this technique is controversial and may lead to a higher level of recurrence if tumor is left behind, our patient had no local recurrence on imaging at 4 years postoperatively. Despite a high grade lesion with an infiltrating aspect into the adjacent organs, the resection margins were negative except at the thyroid isthmus. With a 4-year follow-up without local recurrence, it appears to us that a disfiguring resection would not have been necessary in the management of our patient.

The first step to performing an optimal resection, while leaving in place as much healthy tissue as possible, is an exhaustive preoperative assessment of the lesion. This assessment includes a neck and chest CT scan, a neck and chest MRI, as well as a panendoscopy. The goal of the panendoscopy is to detect any esophageal or tracheal transfixing lesions that may contraindicate the surgical procedure or impose to perform tracheal resection. The American Thyroid Association, in its 2012 and 2021 recommendations on the management of anaplastic thyroid cancer (which is also aggressive), recommended routine preoperative laryngoscopy and esophagoscopy for a rapidly progressive mass in the neck [15, 16]. In the case of a rapidly evolving infiltrative lesion (regardless of the type of cancer), we believe that a panendoscopy is essential.

Although there is no consensus in the literature about the management of patients with primary thyroid sarcoma, the first therapeutic choice is surgical excision. Indeed, Guarda et al. in their 2018 review of liposarcoma showed that all patients had undergone surgical resection. Surgical resection has better results than radiation therapy alone. Gerry et al. [17] showed that patients with head and neck liposarcoma receiving only radiation therapy had a significantly worse outcome than patients receiving either surgical resection or surgery plus adjuvant radiation therapy. Davis et al. [18] recommended surgery with negative margin resection as the treatment of choice for liposarcoma of the head and neck. Regular and frequent follow-up after surgery is an option even for patients with well-differentiated or myxoid liposarcomas with negative margins without local extension. Adjuvant chemo and/or radiation therapy should be considered for high-grade tumors, positive margins, large tumors, local extension, and tumors in complex anatomic areas. In sarcomas, the improvement in locoregional control and overall survival achieved by the addition of radiation therapy has been shown to be related to tumor grade and narrow surgical margins [19]. Since it is often difficult to obtain wide surgical margins in the neck, not surprisingly, postoperative radiation therapy has been shown to decrease local recurrence [20].

## Conclusions

Thyroid liposarcoma is a rare lesion of the thyroid. Its management requires an exhaustive workup followed by an en bloc resection of the lesion. Depending on the histology, postoperative radiation therapy may or may not be necessary.

## Supplementary Material

Supplementary Material
