# Supplementary material for: Thyroid liposarcoma: a case report
Source: Innov Surg Sci. 2022 Oct 14;7(3-4):133–7. doi: 10.1515/iss-2021-0037 (PMC9742268; doi:10.1515/iss-2021-0037)
Supplement: Supplementary file 1 — Supplementary Material [file j_iss-2021-0037_suppl.pdf]

Reviewer Assessment

M. Gerard et al.: Thyroid liposarcoma: a case report

Reviewers' Comments to Original Submission

Reviewer 1: Stefanou, Christos

Date received: 07-Jun-2022
Reviewer recommendation: **Accept in present form**
Reviewer overall scoring: **Excellent**

Assessment Form scores: 5 = High/Yes; 3 = Medium/Adequate; 1 = Low

Is the subject area appropriate for the journal	5				
Does the title clearly reflect the paper's content?	5				
Does the abstract clearly reflect the paper's content	5				
Do the keywords clearly reflect the paper's content?		4			
Does the introduction present the problem clearly?	5				
Are the results/ conclusions justified?	5				
How comprehensive and up-to-date is the subject matter presented?	5				
How adequate is the data presentation?	5				
Are units and terminology used correctly?	5				
Is the number of cases adequate?			3		
Are the experimental methods/ clinical studies adequate?	5				
Is the length appropriate in relation to the content?	5				
Does the reader get new insights from the article?	5				
Please rate the practical significance.	5				
Please rate the accuracy of methods.		4			
Please rate the statistical evaluation and quality control.		4			
Please rate the appropriateness of the figures and tables.	5				
Please rate the appropriateness of the references.	5				
Please evaluate the writing style and use of language.	5				
Please judge the overall scientific quality of the manuscript.	5				
Are the methods used worthy of reproduction in greater deal?	Yes				
Would you be willing to review a revision of this manuscript?	No				

Comments to author: A very rare case report. Very well described.
Well done with the work up and the treatment.

Reviewer 2: anonymous

Date received: 29-Apr-2022
Reviewer recommendation: **Return to author for minor modifications**
Reviewer overall scoring: **Medium**

Assessment Form scores: 5 = High/Yes; 3 = Medium/Adequate; 1 = Low

Is the subject area appropriate for the journal		4			
Does the title clearly reflect the paper's content?	5				
Does the abstract clearly reflect the paper's content	5				
Do the keywords clearly reflect the paper's content?	5				
Does the introduction present the problem clearly?	5				
Are the results/ conclusions justified?		4			
How comprehensive and up-to-date is the subject matter presented?			3		
How adequate is the data presentation?			3		
Are units and terminology used correctly?			3		
Is the number of cases adequate?					1
Are the experimental methods/ clinical studies adequate?		4			
Is the length appropriate in relation to the content?		4			
Does the reader get new insights from the article?			3		
Please rate the practical significance.			3		
Please rate the accuracy of methods.		4			
Please rate the statistical evaluation and quality control.					1
Please rate the appropriateness of the figures and tables.			3		
Please rate the appropriateness of the references.		4			
Please evaluate the writing style and use of language.		4			
Please judge the overall scientific quality of the manuscript.		4			
Are the methods used worthy of reproduction in greater deal?					
Would you be willing to review a revision of this manuscript?	Yes				

Comments to author: Dear authors,
this is a nicely presented case, which is truly worthwhile to be published.
However, I have one major concern: this refers to the histology. You describe that the sarcoma invades the thyroid, so my question is: is it truly a liposarcoma of the thyroid itself or is it a liposarcoma originating somewhre else and just invading the thyroid? This needs definitely to be clarified and I highly recommend an additional figure which clearly shows the histology that it is truly a thyroid liposarcoma. This should be witnessed by the pathologist who did the examination. He or she should then be a coauthor of the paper.
I am glad to review the paper again, once it is revised.

Authors' Response to Reviewer Comments

Date received: 17-Jul-2022

Response to reviewer 1

Thank you for your comment and your pertinent remark. The pathological evaluation showed that the lesion was invading the thyroid. The origin of the lesion seems to be the peri-thyroidal adipose tissue (this is stated in the report). I think, as you say, that this may not be a ”true” liposarcoma of the thyroid itself. Unfortunately, we do not have any images to show. The pathologist who read the slides is already on the author-list (Dr Myriam DECAUSSIN-PETRUCCI).

Response to reviewer 2

Thank you for your comment and your pertinent remarks.

Comments by the Editor-in-Chief to Revised Submission

All reviewer comments were addressed adequately and changes made to the manuscript accordingly and the manuscript should be published in its present stage.
